# Electrospun Biodegradable α-Amino Acid-Substituted Poly(organophosphazene) Fiber Mats for Stem Cell Differentiation towards Vascular Smooth Muscle Cells

**DOI:** 10.3390/polym14081555

**Published:** 2022-04-11

**Authors:** Meng Wang, Shigang Lin, Kibret Mequanint

**Affiliations:** Department of Chemical and Biochemical Engineering, The University of Western Ontario, London, ON N6A 5B9, Canada; mwang529@uwo.ca (M.W.); slin45@uwo.ca (S.L.)

**Keywords:** biodegradable poly(organophosphazenes), human mesenchymal stem cells, vascular smooth muscle cells, vascular tissue engineering, electrospinning

## Abstract

Mesenchymal stem cells, derived from human-induced pluripotent stem cells (iPSC), are valuable for generating smooth muscle cells (SMCs) for vascular tissue engineering applications. In this study, we synthesized biodegradable α-amino acid-substituted poly(organophosphazene) polymers and electrospun nano-fibrous scaffolds (~200 nm diameter) to evaluate their suitability as a matrix for differentiation of iPSC-derived mesenchymal stem cells (iMSC) into mature contractile SMCs. Both the polymer synthesis approach and the electrospinning parameters were optimized. Three types of cells, namely iMSC, bone marrow derived mesenchymal stem cells (BM-MSC), and primary human coronary artery SMC, attached and spread on the materials. Although L-ascorbic acid (AA) and transforming growth factor-beta 1 (TGF-β1) were able to differentiate iMSC along the smooth muscle lineage, we showed that the electrospun fibrous mats provided material cues for the enhanced differentiation of iMSCs. Differentiation of iMSC to SMC was characterized by increased transcriptional levels of early to late-stage smooth muscle marker proteins on electrospun fibrous mats. Our findings provide a feasible strategy for engineering functional vascular tissues.

## 1. Introduction

Biodegradable poly(organophosphazenes), substituted with α-amino acids and hereafter abbreviated as PαAPz, are important biomaterials for drug delivery and tissue engineering applications [1,2,3,4]. Unlike biodegradable polyesters (e.g., poly(lactide), poly(glycolide), and their copolymers), PαAPz releases non-acidic and buffering degradation products comprised mainly of phosphate, ammonia, and the corresponding side groups [5,6]. Several previous studies investigated PαAPz primarily, for the regeneration of musculoskeletal tissues, and demonstrated their ability to support osteoblast adhesion and proliferation with minimal inflammatory responses and enhanced bone growth [1,2,3,7,8,9]. To introduce porosity to bone tissue engineering PαAPz scaffolds, either dynamic solvent sintering of pre-formed microspheres [10,11] or electrospinning of PαAPz blended with nanohydroxyapatite [12] have been studied. In one study [13], where a PαAPz was electrospun for bone tissue engineering, the fiber diameter was large (~55 µm) making it challenging for osteoblasts to see the true 3D topography of the fibers. In addition to bone cell culture, some studies investigated electrospun PαAPz for their potential use of an endothelial monolayer cell culture [14,15]. In general, electrospun scaffolds are preferred in comparison to other forms of porous scaffolds to better mimic the fibrous extracellular matrix [16,17].

While the above-mentioned studies demonstrated the versatility of PαAPz, primarily in bone regeneration, their utility in vascular tissue engineering has not been studied. Vascular smooth muscle cells (VSMCs) are the major cellular components to engineer vascular tissues. Since VSMCs are particularly sensitive to acidic degradation products, we believe that PαAPz that degrade into non-acidic by-products could be excellent biomaterials for smooth muscle tissue engineering. Considering that harvesting primary VSMCs from patients is not feasible due to their inaccessible anatomical location (e.g., coronary artery), mesenchymal stem cells (MSCs) are ideal cell sources for vascular tissue engineering. MSCs are multipotent cells commonly characterized by their ability to differentiate into cell types of mesodermal origin [18,19,20,21,22,23]. MSCs can be isolated from many adult tissue sources in a non-invasive manner [24,25]; however, their self-renewal and differentiation potential is dependent on the cell source of isolation [26]. It may also be affected by donor aging and environmental stresses from in vitro cultivation [27]. To circumvent these challenges, the use of iPSCs for generating MSCs is beneficial to preserve their high differentiation potential [28,29].

MSCs can be differentiated into VSMCs to engineer vascular tissues for morphogenesis and functional studies. VSMCs express smooth muscle phenotypic marker genes and proteins, such as α-SMA and calponin, which are early and mid-stage differentiation markers, as well as myosin heavy chain (MHC), smoothelin (SMTN), and smoothelin-B, which represent late-stage differentiation stages. Expression of early and mid-stage markers only indicates progression towards a smooth muscle lineage and are also expressed in other cells; however, the late-stage marker proteins MHC and SMTN [30,31] are exclusively expressed in mature contractile VSMCs. In view of this, biomaterials designed for vascular tissue engineering must promote the differentiation of MSCs to VSMCs by providing mechanical and topological cues [32]. Although MSC differentiation to vascular smooth muscle cells on electrospun fibers have recently been reported, the fibers were prepared from a non-degradable polyacrylonitrile [33] or else the fibers were used only to deliver a biochemical factor [34].

The objectives of this study were twofold. First, we aim to synthesize electrospinnable PαAPz by modifying the synthesis procedure. This is important since most PαAPz are generally difficult for producing electrospun fibrous mats without being blended with other polymers, and only a few previous studies attempted doing this. Secondly, we aim to use the fibrous mats for the differentiation of iPSC-derived MSCs towards vascular smooth muscle cells.

## 2. Experimental

### 2.1. Materials

Hexachlorocyclotriphosphazene (HCCP) was obtained from Sigma Aldrich (Milwaukee, WI, USA) and stored in the desiccator until used. Anhydrous tetrahydrofuran (THF) and glass distilled hexanes-190 were acquired from Caledon Labs (Georgetown, ON, Canada). Triethylamine (NEt_3_), chloroform (CF), and dimethyl sulfoxide (DMSO) were purchased from Sigma Aldrich (Milwaukee, WI, USA). L-alanine ethyl ester hydrochloride (H-Ala-Oet · HCl) and L-phenylalanine ethyl ester hydrochloride (H-Phe-Oet · HCl) were from Alfa Aesar (Ward Hill, MA, USA) and stored in the fridge. Krytox Performance Lubricant GPL207 high-temperature grease was purchased from DuPont (Wilmington, DE, USA). Unless specified otherwise, all chemicals and solvents were used as received.

### 2.2. Synthesis of PαAPz

Polydichlorophosphazene (PDCP) was prepared using thermal ring-opening polymerization (TROP) with three different approaches, namely: grease sealing, flame sealing, and HCCP recrystallization, followed by flame sealing. In the grease sealing approach, approximately 1 g HCCP was added into a dry glass reactor fitted with a stopper, and argon was purged to displace any residual air on the walls of the reactor. For the flame sealing and recrystallization (FS-R) approach, HCCP was first recrystallized by vacuum sublimation at 100 °C and 20 mmHg before use. Following recrystallization, approximately 2 g of HCCP was added into a glass ampoule, connected to a vacuum line, and flame-sealed using a propane torch. Alternatively, the vacuum step could be replaced by filling the ampoules with dry argon, followed by flame sealing. The sealed ampoules were placed in the oven at 230 °C for 72 h. The samples were recovered from the ampoules, dissolved in THF, and purified by precipitating in hexanes three times to remove the unreacted HCCP and crosslinked PDCP. PDCP recovery and purification were similar for grease sealing and flame sealing.

### 2.3. Macromolecular Substitution

The macromolecular substitution reaction was carried out by a one-step approach at room temperature, as described before [35]. Briefly, after flame drying, a 50 mL flask of either H-Ala-OEt · HCl (1.70 g, 11.1 mmol, 2.6 equiv.) or H-Phe-OEt · HCl (2.16 g, 11.1 mmol, 2.6 equiv.) was added into the reaction flask, and then, the flask was sealed with a rubber septum. Next, 10 mL of THF and 4 mL of triethylamine were injected into the flask through the septum. Then, PDCP (0.50 g, 4.3 mmol of N = P-Cl_2_ units) obtained from the TROP was dissolved in 10 mL of anhydrous THF and injected into the reaction flask through the septum. The mixture was stirred for 72 h, filtered to remove the insoluble salt, and the product was dissolved in anhydrous THF and purified by repeated precipitation in hexanes. The PαAPz polymers were then dried in a vacuum and stored at 4 °C.

### 2.4. PDCP and PαAPz Characterization with ^31^P-NMR and ^1^H-NMR

Nuclear Magnetic Resonance (NMR) spectroscopy was performed on a Varian INOVA 400 MHz spectrometer (^1^H 400.1 MHz, and ^31^P{1H 161.8 MHz, Varian Canada Inc., Mississauga, ON, Canada). Chemical shifts are reported in parts per million (ppm). All chemicals are dissolved in chloroform (CDCl_3_) with a concentration of ~40 mg/mL. Chemical shifts were relative to chloroform at δ = 7.27 ppm.

### 2.5. Preparation of PαAPz Thin Films

PαAPz was dissolved in THF to form a 1 wt.% solution. Then 67.8 μL of the solution was dipped onto a glass coverslip with a diameter of 12 mm. After drying, all of the film was sterilized under the UV light for 30 min and pre-treated with Hank’s Balanced Salt Solution (HBSS) overnight.

### 2.6. Electrospinning of PαAPz

The electrospinning equipment consists of 0.5 mL glass syringes, blunt-tip 22-gauge stainless steel needles, high voltage DC power supply (7–20 kV, ES30P, Gamma High Voltage, Ormond Beach, FL, USA), syringe pump (KD101, KD Scientific, Holliston, MA, USA), and rotating collector. The PαAPz were electrospun at various concentrations and spinning parameters. The morphology of the PαAPz fibrous mat was evaluated using SEM (S-2600N Hitachi, Tokyo, Japan). Fibrous samples were sputter-coated with gold/palladium (K550X, sputter coater, Emitech Ltd., Ashford, UK) and scanned at a working distance of 9 mm and a constant accelerating voltage of 5 kV. Analysis of the SEM was performed with ImageJ software (NIH, Bethesda, MD, USA).

### 2.7. Cell Culture Studies on PαAPz and Smooth Muscle Cell Differentiation

Mesenchymal stem cells derived from induced pluripotent stem cells (iPSCs), hereinafter named iMSCs (kindly donated by Dr. Dale Laird, Western University, Canada), and human bone marrow-derived mesenchymal stem cells (BMMSCs, Lonza, Walkersville, MD, USA; PT-2501) were used. iMSCs were grown on gelatin (Sigma-Aldrich Canada Co., Oakville, ON, Canada) coated dishes in mesenchymal stem cell expansion media (MSCEM, Cedarlane Labs, Burlington, ON, Canada; HMSC.E.MEDIA-450) supplemented with 10% fetal bovine serum (FBS), 1% l-glutamine, and 1% penicillin/streptomycin (all from Fisher Scientific, Whitby, ON, Canada). Media were changed every other day. Human coronary artery smooth muscle cells (HCASMCs, Lonza, Cohasset, MN, USA. CC-2583) were maintained in Smooth Muscle Cell Growth Medium 2 BulletKit (SmGM-2, Lonza, Cohasset, MN, USA). Cells between passage numbers 4–11 were used for experiments. Cells were cultured in humidified incubators at 37 °C and 5% CO_2_. Differentiation of stem cells to smooth muscle cell lineage was induced using L-ascorbic acid (L-AA, Sigma-Aldrich Canada Co., Oakville, ON, Canada) and transforming growth factor (TGF-β1, R&D Systems, Minneapolis, MN, USA) in high glucose DMEM, modified based on reported procedures [36]. Briefly, iMSCs were plated either on gelatin-coated dishes for 2D study (2 × 10^4^ cells/cm^2^ in 6-cm Corning cell culture dish) or on gelatin-coated electrospun fibrous mats for 3D study (20 × 10^4^ cells per mat with a size of 2 cm × 2 cm) and pre-cultured in MSCEM to reach 70% of confluence, followed by changing culture media to differentiation condition (DC), composed of DMEM supplemented with 1% FBS plus 82.5 μg/mL [37] L-AA or 2 ng/mL TGF-β1. The induced smooth muscle-like cells were characterized by the detection of smooth muscle marker genes and proteins using qRT-PCR, Western blot analysis, as well as immunofluorescence staining. All fibrous mats were coated with 0.1% gelatin at 37 °C for 1 h before cell seeding. Non-induced iMSCs were grown in regular MSCEM as the undifferentiated growth control (GC).

### 2.8. Quantitative Real-Time qPCR and Western Blot Analysis

Total RNA was extracted from cells using TRIzol™ Reagent (Life Technologies Inc., Burlington, ON, Canada) following the manufacturer’s instructions. One μg of total RNA was reverse transcribed into complementary DNA (cDNA) using the Promega™ Random Hexamers protocol (Fisher Scientific, Whitby, ON, Canada). qRT-PCR was conducted in four repeats using a CFX96™ Real-Time System (C1000 Touch Thermal Cycler; Bio-Rad, Mississauga, ON, Canada), and human genes of interest were determined with SsoAdvanced Universal SYBR^®^ Green Supermix (Bio-Rad, Mississauga, ON, Canada), according to the recommended procedures. The sequences of primers are presented in Table 1. The results were analyzed with the comparative threshold cycle method and normalized with human 18S as an endogenous reference.

Western blotting was performed to evaluate the expression levels of specific smooth muscle marker proteins. Cell lysates were separated via SDS-PAGE, transferred to nitrocellulose membrane, and immunoblotted using the following primary antibodies: SM-MHC (rabbit, 1:1000; Alfa Aesar (Haverhill, MA, USA), BT-562), SMTN-B (rabbit, 1:1000; Santa Cruz (Dallas, TX, USA), sc-28562), and β-Tubulin (mouse, 1:250; ThermoFisher Scientific (Waltham, MA, USA), MA5-11732). Primary antibody labeling was detected using HRP-conjugated goat anti-rabbit or anti-mouse secondary antibodies, and the ECL detection system.

### 2.9. Immunofluorescence Microscopy

Cells were fixed in 10% formalin for 10 min at ambient temperature, followed by three washes in PBS. All samples were permeabilized with 0.1% Triton X-100 for 10 min, and then, they were blocked in 2% BSA, with 22.52 mg/mL of glycine in PBS, for 30 min at ambient temperature. Samples were labeled with the following antibodies overnight at 4 °C: α-SMA (mouse, 1:100; Santa Cruz (Dallas, TX, USA), sc-32251), SM-MHC (rabbit, 1:100; Alfa Aesar (Haverhill, MA, USA), BT-562), SMTN-B (rabbit, 1:100; Santa Cruz (Dallas, TX, USA), sc-28562), and SMTN (rabbit, 1:100; Santa Cruz (Dallas, TX, USA), sc-166292). Primary antibody binding was detected using Alexa Fluor^®^ 555 goat anti-rabbit IgG or Alexa Fluor^®^ 488 goat anti-mouse IgG as secondary antibodies (1:300) addition with Alexa Fluor^®^ 568- or Alexa Fluor^®^ 488 phalloidin (1:100 dilution, all from Life Technologies, Burlington, ON, Canada), in some cases. The cells were counterstained with 4′6-diamidino-2-phenylindole (DAPI, 300 nM in PBS, Life Technologies, Burlington, ON, Canada) for labeling nuclei. Coverslips were mounted on microscope slides with PermaFluor™ Mounting Medium (Fisher Scientific™, Whitby, ON, Canada) and sealed with clear nail enamel. Images were taken with a Zeiss LSM 800 confocal microscope (Zeiss, Toronto, ON, Canada) equipped with 10×/25× (water) lenses and analyzed using ZEN 3.1 (blue edition) software.

### 2.10. Statistical Analysis

Data are presented as mean ± SD unless otherwise indicated. At least three independent experiments were analyzed by one-way ANOVA with Student’s two-tailed independent sample *t* test. All analyses were done using GraphPad 5.0 statistical software (GraphPad Software) and significance was assigned for *p* < 0.05

## 3. Results and Discussion

### 3.1. PαAPz Synthesis and Characterization

**Figure 1:**
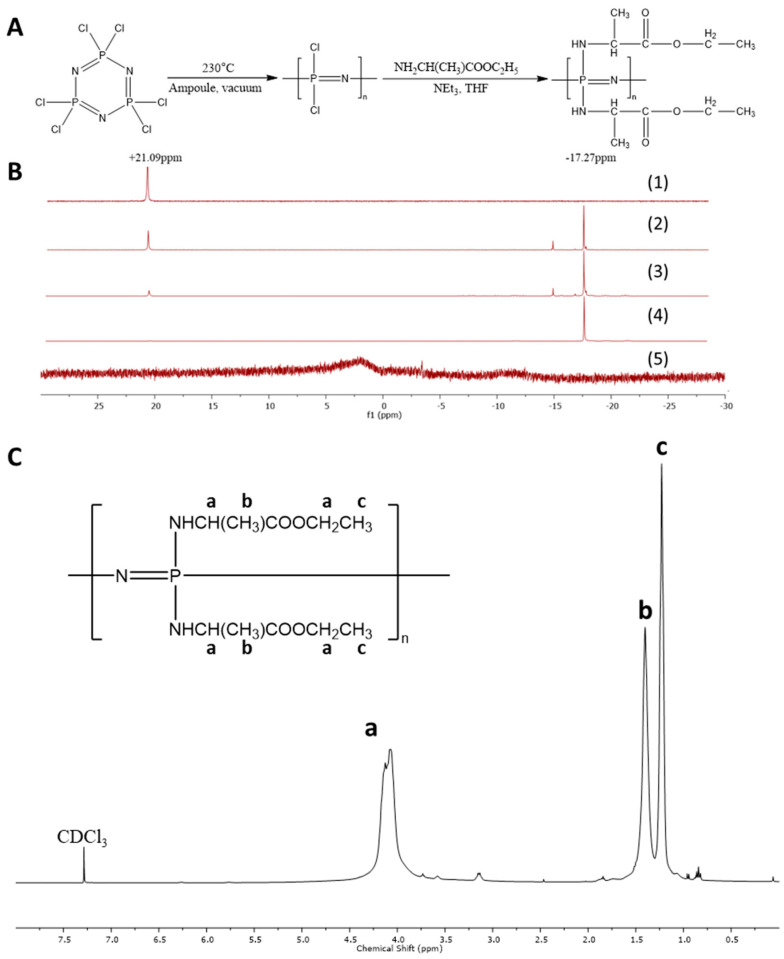
(**A**) Synthesis scheme of PαAPz-A. (**B**) ^31^P NMR of (1) HCCP, (2) Crude PDCP from GS method, (3) Crude PDCP from FS, and (4) purified PDCP, (5) PαAPz-A. (**C**) ^1^H NMR of PαAPz-A.

The synthesis of PαAPz is a two-step procedure. In the first step, the thermal ring-opening polymerization of HCCP at high temperature (ca. 230 °C) produces PDCP. In the second step, the chlorine atoms attached to the central phosphorous are replaced with side chains via a macromolecular substitution reaction at room temperature (Figure 1A). The thermal ring-opening reaction requires a strict anhydrous environment and specialized equipment. In this study, we attempted to simplify the complexity involved in the thermal ring-opening reaction while, at the same time, reducing the undesirable hydrolysis and crosslinking. Since the primary objective in the ring-opening reaction is to avoid moisture, we attempted six different approaches (Table 2). In the first approach (GS method), we used high-temperature fluorinated grease as a potential moisture barrier when sealing the reaction content using a glass stopper [35]. The main component of this grease is perfluoroalkyl ether, which was used for food packaging and proved to have the ability to provide moisture resistance [38]. With the GS method, the product crosslinked after 25 h reaction, so the reaction was stopped at this time. In a modification of the GS procedure, we purged the glass reaction vial with argon and sealed it with the same grease and glass stopper, with the expectation that the argon may displace any residual moisture/air present around the walls of the glass vial due to its high density (GS-Ar method). Indeed, the conversion of HCCP to PDCP increased from 54% to 86% when argon was used to displace air, although it was crosslinked after 54 h of reaction. For the remaining four thermal ring-opening reaction methods, flame sealing of the glass ampoule was used. Thus, flame sealing (FS), flame sealing using argon (FS-Ar), flame sealing under vacuum (FS-vacuum), and recrystallization of HCCP followed by flame sealing under vacuum (R-FS-vacuum) were explored. Both FS-vacuum and R-FS-vacuum methods produced PDCP that was not crosslinked during 92 h of reaction at 230 °C. In addition, the conversion of HCCP to PDCP increased to 95%; thus, FS-vacuum and R-FS-vacuum methods were taken as appropriate methods to produce linear PDCP for the subsequent macromolecular substitution with α-amino acid esters. The thermal ring-opening polymerization time of HCCP to produce PDCP is up to 120 h, and the goal is to get very high conversions without crosslinking. Since the reaction occurs in the melt, the occurrence of crosslinking is easily detected by visually inspecting if the melt still flows when the ampoule is held upside down.

^31^P-NMR spectra for HCCP, linear PDCP, PαAPz, and ^1^H-NMR spectrum of poly [bis (ethyl alanato) phosphazene] (PαAPz-A) are shown in Figure 1B,C. Consistent with the literature, HCCP shows a single peak at 21.09 ppm, and PDCP, which is the product from the thermal ring-opening reaction after purification by precipitation, showed a singlet at approximately −17 ppm. In Figure 1B, representative ^31^P-NMR spectra of PDCP from grease sealing and flame sealing methods before purification are also shown (B2, B3).

In the ^31^P-NMR spectrum of PαAPz, after the macromolecular substitution with alanine ester, the peak at −17.27 ppm, representing the phosphorous atom on PDCP, disappeared and there is only a small peak at +1.41 ppm (Figure 1B5) which lies between the reported values of +1.2 to +1.9 for PαAPz [39]. The complete disappearance of the peak at −17.27 ppm means that most, if not all, Cl atoms in the PDCP have been replaced. In the ^1^H-NMR, all the expected peaks for the ethyl ester of alanine are shown, which confirmed the successful substitution. As reported before [35], the hydrogen connected to the amino group was not seen in the ^1^H-NMR spectrum. In addition, the ^1^H-NMR demonstrated that the unreacted amino acid had been fully removed during the precipitation process. In the present study, we used L-alanine and L-phenylalanine as the preferred amino acids for the following two reasons. (i) From our previous study [35], we observed that both of these amino acids produced PαAPz that have comparable molecular weights that could be suitable for electrospinning. (ii) Both of these amino acids are neutral and non-polar, yet the glass transition temperatures (*T*_g_) of the corresponding PαAPz are different. PαAPz-A has a *T*_g_ that is in the range of −20 °C to −10 °C making it flexible at a cell culture temperature of 37 °C, whereas PαAPz-F has a *T*_g_ above 37 °C [35,40] that could display some brittleness during cell culture handling and subsequent staining.

### 3.2. Electrospinning of PαAPz

**Figure 2:**
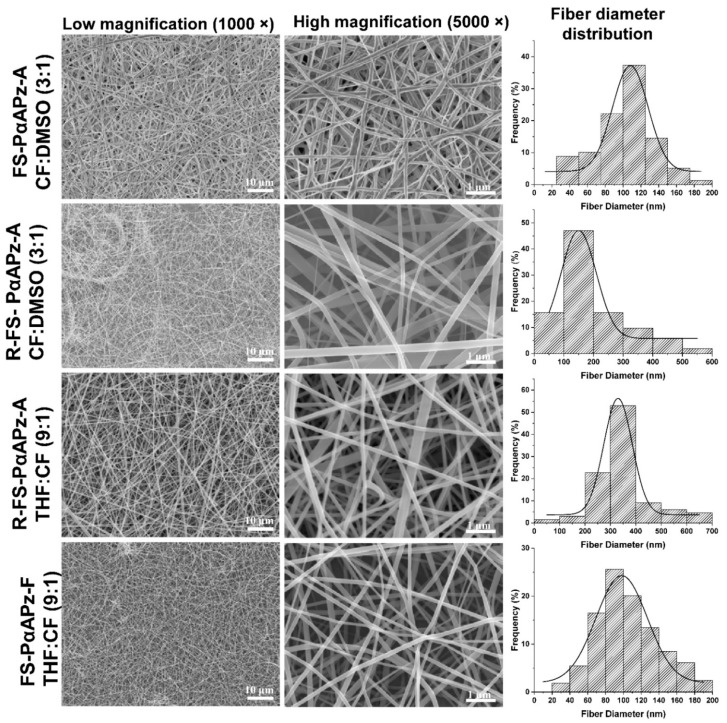
SEM images of electrospun PαAPz derived from L-phenylalanine and L-alanine, as well as the corresponding histogram, showing the fiber diameter distribution. For each SEM image, the PDCP synthesis method (FS—flame sealing; R-FS: recrystallization followed by flame sealing) and the optimum solvent ratio are shown. Other electrospinning parameters are listed in Table 3.

Although PαAPz have been used for drug delivery studies in solid and film forms, electrospinning allows for the formation of fibers that can be utilized for tissue engineering. However, standalone fiber formation from PαAPz is often challenging except for a few reports [15,41]. Therefore, we have studied a wide range of electrospinning parameters and solvent combinations to produce fibrous mats (Table 3). The PαAPz-A and PαAPz-F prepared in the current study were electrospinnable from a solution of THF or CF, but that depended on the method of the PDCP preparation. Although PαAPz-A and PαAPz-F were successfully synthesized from PDCP that was prepared by all the methods listed in Table 2, only PDCP from FS-Vacuum and R-FS-Vacuum methods resulted in PαAPz that could be consistently electrospun. PDCP prepared by grease sealing resulted in a corresponding PαAPz that led to electrosprayed particles instead of fibers attributed to hydrolysis of PDCP during its synthesis and subsequent lower molecular weight of PαAPz. It is known that lower molecular weight polymers often produce electrosprayed particles rather than continuous fibers [42,43,44] due to the lower solution viscosity that makes the Taylor cone unstable during electrospinning [45]. Figure 2 shows the SEM images of the electrospun PαAPz fiber mats that were synthesized from PDCP and, in turn, were prepared from the FS method, with both low magnification (large scan area) and high magnification (small scan area). The fibers were free from defects and had diameters below 0.7 µm with averages between 0.1 µm and 0.3 µm.

For the PαAPz-A polymers, the CF:DMSO solvent combination was used to electrospin them. When the CF alone was used, the needle tip was clogged due to the rapid evaporation of the solvent, while the fibers collected also had defects due to beading. Thus, 25% DMSO was added to address both challenges. As an alternative, it was also possible to electrospin PαAPz-A polymers from THF:CF (9:1) ratio using the indicated parameters of Table 3. It is noteworthy that PαAPz-F polymers were only electrospun in the THF:CF (9:1) ratio. The fiber size distribution appeared to be influenced by the PDCP synthesis approach rather than the solvent combination since the recrystallization of HCCP, followed by flame sealing, produced PαAPz that have slightly higher solution viscosity, at the same concentration, compared with those PαAPz that were prepared from PDCP without HCCP recrystallization.

### 3.3. Short-Term In Vitro Degradation Study

**Figure 3:**
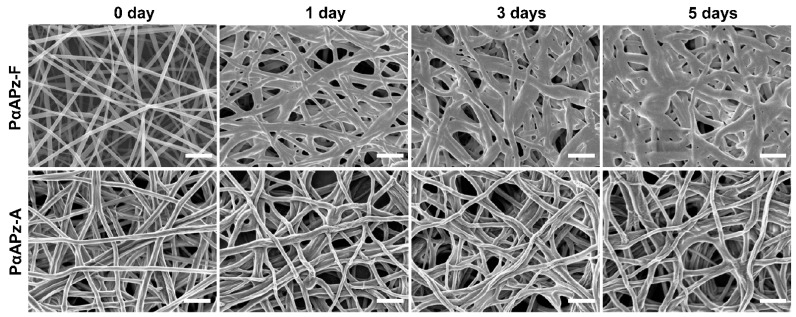
SEM images showing the short-term in vitro degradation study of electrospun mats fabricated from PαAPz, derived from the amino acids L-phenylalanine (PαAPz-F) and L-alanine (PαAPz-A). PαAPz-A fibers lost their porous morphology faster than PαAPz-A fibers. Scale bar = 1 μm and is applicable to all images.

PαAPz are biodegradable polymers due to the presence of α-amino acid residues. While this is a desired property for tissue engineering, rapid degradation (within 3–5 days) of the electrospun fibers is a disadvantage, since infiltration of seeded cells into the porous mats will be minimal. Cell infiltration into electrospun fibers is generally challenging, and early degradation exacerbates it as the fibers fuse and thus limit infiltration. To gain an insight into the early degradation of the fiber mats, samples were incubated in PBS at 37 °C for up to 5 days. As presented in Figure 3, the fiber morphologies of PαAPz-A did not change during the 5 days incubation; however, progressive fiber thickening, flattening, and eventual fusion was observed for the PαAPz-F. After five days of incubation, the fibrous mats lost their porosity considerably, presumably due to the propensity of L-phenylalanine to hydrolysis compared with alanine. In addition, PαAPz-F mats displayed some brittleness, making them difficult for cell culture handling and subsequent staining. All cell culture experiments were therefore conducted on PαAPz-A.

### 3.4. Cell Adhesion and Morphology on PαAPz-A Films

**Figure 4:**
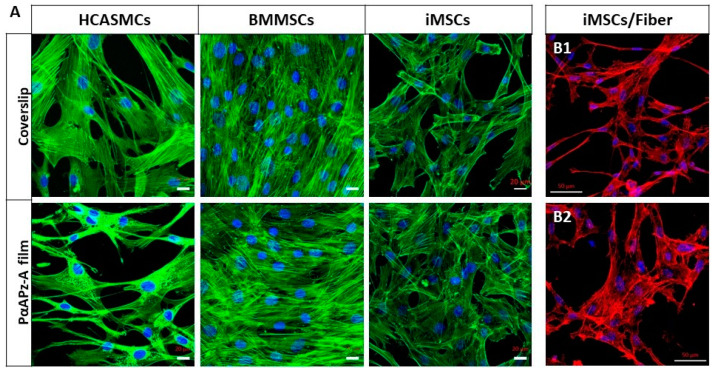
(**A**) Fluorescence images of different cell types on PαAPz-A films and on coverslips. After 3 days of culture, F-actin is labeled by Phalloidin in green, and nuclei are labeled by DAPI in blue. Scale bar = 20 μm. (**B1**,**B2**) Fluorescence images of iMSCs after 2 days (**B1**) and 4 days (**B2**) of culture on PαAPz-A electrospun fibrous mats. Red channel is F-actin, and nuclei are labeled by DAPI in blue. Scale bar = 50 μm.

To investigate in vitro cell adhesion and morphology, three distinct cell types were seeded on PαAPz-A films over a three-day period. Since primary human vascular cells are not readily available, we evaluated the potential of obtaining them by differentiating bone marrow-derived mesenchymal stem cells (BMMSC) and mesenchymal stems cells (iMSC) from induced pluripotent stem cells. Human coronary artery smooth muscle cells (HCASMC) were used as control. Confocal microscopy images shown in Figure 4A demonstrated that all three types of cells attached and spread on the surface of PαAPz-A films after three days of culture and were morphologically indistinguishable from cells cultured on the glass coverslips. All three types of cells showed abundant F-actin expression; however, BMMSCs had a better attachment, as judged by the dense cell layer observed. As we have initially seeded the same number of cells, and the culture time is only three days, the observed high cell density in BMMSC is related to high retention rather than cell growth. Although cell retention is high for BMMSC, their application as a cell source is not as robust compared with iMSC since they require invasive procedures to obtain them, and they have replicative senescence during expansion [46]. Compared to primary HCASMC, iMSC’s retention on the PαAPz-A film was high, suggesting that these cells were viable. Thus, iMSC attachment and growth were further evaluated on the electrospun fibrous mats by F-actin fluorescence staining after culturing iMSCs on PαAPz-A fibrous mats for 2 days (Figure 4(B1)) and 4 days (Figure 4(B2)). As seen from these confocal images, iMSCs were well-spread on the fibrous mats, characterized by well-defined F-actin stress fibers with a typical small cell body that is of long and thin morphology, which is characteristic of stem cells, suggesting that the scaffold microenvironment was favorable for cell attachment and growth.

### 3.5. Differentiation Potential of iMSCs towards Smooth Muscle Phenotype

**Figure 5:**
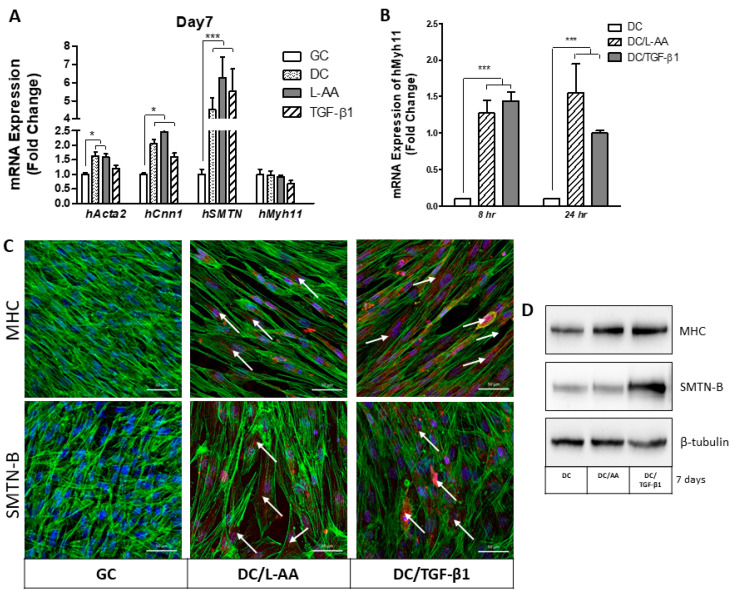
Differentiation potential of iMSCs towards vascular smooth muscle lineage in conventional 2-D cultures. (**A**) iMSC mRNA expression of *hActa2*, *hCnn1*, *hSMTN*, and *hMyh11* was analyzed using Quantitative real-time polymerase chain reaction (qRT-PCR) after cell treatment with L-AA and TGF-β1 for 7 days. GC stands for regular growth condition of iMSCs, and DC stands for differentiation inducible condition by exchanging culture media to DMEM with 1% FBS. (**B**) mRNA expression of *hMyh11* by iMSCs after 8 h and 24 h of cultivation under DC. (**C**) iMSCs were cultured on coverslips, under differential conditions, for 7 days, MHC and SMTN-B were detectable with either AA or TGF-β1 pre-treatment. Green channel is F-actin, and Red is MHC/SMTN-B. Nuclei are labeled by DAPI in Blue. Scale bar = 50 μm. (**D**) Western blot analysis revealed that vascular smooth muscle contractile marker SMTN-B was up-regulated by pre-treatment with TGF-β1, whereas MHC was slightly up-regulated by either L-AA or TGF-β1 pre-treatment. * *p* < 0.05; *** *p* < 0.001.

After establishing that PαAPz-A fibrous mats were able to support attachment and spreading of iMSCs, we next investigated if these cells have the differentiation potential towards the vascular smooth muscle phenotype. We chose iMSCs because BMMSCs have a relative ease to undergo osteogenic and adipogenic differentiation compared to vascular differentiation, which is more complex [47,48,49]. Although direct differentiation of partially induced pluripotent stem cell to a VSMC has been reported [50], those obtained from a fully induced pluripotent cells via a mesenchymal precursor cells are the most established method [51,52,53]. To determine the differentiation capacity of iMSCs into smooth muscle phenotype, the expression of smooth muscle-specific marker genes and proteins was analyzed under 2D conventional tissue culture conditions, and results are shown in Figure 5. iMSCs were plated on gelatin-coated culture dishes in stem cell culture media. Once the cells reach 70% of confluency, the culture media were changed to the following for seven days: (i) differentiation culture (DC) media; (ii) DC + 82.5 μg/mL L-Ascorbic acid (L-AA); (iii) DC + 2 ng/mL TGF-β1. A regular growth condition (GC) was used as a control. As shown in Figure 5A, DC media was able to significantly upregulate the expression of smooth muscle marker gene *Acta2* (*p* < 0.05), *Cnn1* (*p* < 0.05), and *SMTN* (*p* < 0.001) but not the late-stage marker *Myh11* (*p* > 0.05), which suggested that iMSCs possess an intrinsic differentiation capacity towards the smooth muscle phenotype. Upon the addition of AA, the expression levels of *Cnn1* and *SMTN* were further increased.

TGF-β1 is one of the major cytokines that can induce VSMC differentiation of stem/progenitor cells, alone or in combination with other factors. Interestingly, the addition of TGF-β1 into DC media did not provide a synergistic effect on mRNA expression. Moreover, the late-stage marker *Myh11* was less responsive to differentiation inducers after 7 days of culture, which suggested that the *Myh11* marker gene may have been expressed earlier than 7 days. To verify the expression pattern of *Myh11*, iMSCs were differentiated for 8 h and 24 h, using L-AA and TGF-β1, in DC media. As shown in Figure 5B, *Myh11* was notably upregulated by L-AA but was less responsive to TGF-β1. We subsequently performed immunofluorescence staining to detect proteins of MHC and SMTN, two key smooth muscle markers, and the representative confocal images are presented in Figure 5C. Both marker proteins were extensively expressed in DC-treated cells with the addition of L-AA and TGF-β1. Moreover, the expressions of two SMC-specific contractile proteins were detectable, after seven days of culture, using Western blot analysis (Figure 5D). Although MHC protein remains unchanged by either L-AA or TGF-β1 treatment, protein expression of SMTN-B, a specific differentiation marker exclusively expressed in vascular smooth muscle tissues, was upregulated with the addition of TGF-β1.

### 3.6. Differentiation of iMSCs towards Smooth Muscle Phenotype on Electrospun PαAPz-A Fibrous Mats

**Figure 6:**
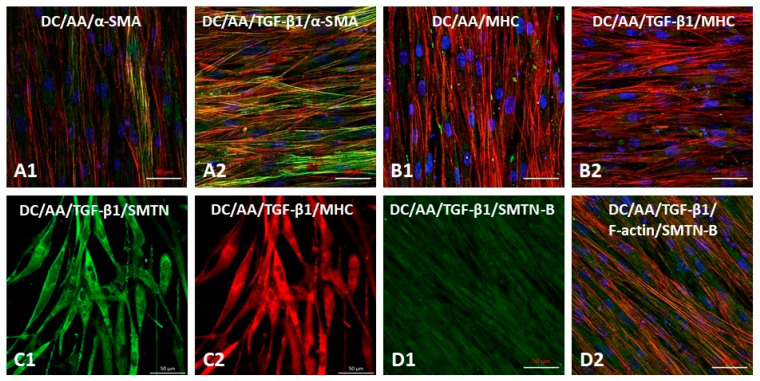
Characteristic analysis of smooth muscle markers by iMSCs seeded on PαAPz-A fibrous mats addition with L-AA or L-AA/TGF-β1 in DC culture media. After 7 days of pre-differentiation, α-SMA (**A1,A2**) and MHC (**B1**,**B2**) were determined by immunofluorescence staining. (**C1**,C**2**) Representative confocal images of SMTN (green) and MHC (red) by iMSCs after 12 days of cultivation. (**D1**,**D2**) Co-localization of SMTN-B (green) and F-actin (red) by iMSCs after 12 days of cultivation. Scale bar = 50 µm.

The preceding cell differentiation results were done on conventional cell culture dishes to rule out the effect of the fiber mat topography. To determine if the microenvironment provided by electrospun PαAPz-A fibrous mats is favorable for vascular differentiation of iMSC, we performed immunofluorescence staining of key vascular smooth muscle markers (α-SMA, MHC, SMTN, and SMTN-B) that represent different stages of smooth muscle differentiation. Stem cell culture on synthetic materials is challenging, as it may transform them into subpopulations with reduced multipotency and fibrotic character; therefore, the microenvironment provided by PαAPz-A fibrous mats with the right physical cues is indispensable for the highly efficient proliferation and differentiation of iMSCs. After seven days of differentiation on PαAPz-A fibrous mats, protein markers α-SMA (green, Figure 6(A1)) and MHC (green, Figure 6(B1)) were observed in L-AA-treated iMSCs, and the fluorescence intensity of early-stage marker protein α-SMA was further enhanced in the presence of TGF-β1 (Figure 6(A2)); however, we did not observe a further increase in the late-stage protein marker MHC (green, Figure 6(B2)) with the addition of TGF-β1. After 12 days of iMSC differentiation, a more contractile phenotype of smooth muscle cells was observed, as verified by immunofluorescence staining of two contractile-specific protein markers, SMTN (Figure 6(C1)) and MHC (Figure 6(C2)), respectively. In addition, SMTN-B is a long isoform of smoothelin that is specifically detectable in vascular smooth muscle tissues and used to discriminate VSMCs from myofibroblasts [54,55]. Consistent with the observation of Western blot analysis in Figure 5D, the co-localization of SMTN-B and F-actin (Figure 6(D1,D2)) confirmed the iMSC differentiation into the smooth muscle phenotype on PαAPz-A fibrous mats. Overall, the immunostaining of SMTN and MHC for cells cultured on the PαAPz-A strongly indicates robust expression compared to conventional cultures shown in Figure 5. Furthermore, differentiated cells were aligned and elongated on the fibrous mats, suggesting directional orientation, presumably due to cues provided by the fiber topography.

PαAPz polymers have been previously studied, almost exclusively, as orthopedic biomaterials, including tendon and ligament [2,3], but their use in vascular smooth muscle differentiation of stem cell tissues has not been reported before. Regarding other vascular cells, to the best of our knowledge, only two previous studies [14,15] reported endothelial cell interaction with PαAPz fibers. Furthermore, standalone electrospinning of PαAPz is challenging, and it is often modified with other polymers such as polycaprolactone [8,35,56,57]. In this study, we showed for the first time that iMSCs could differentiate to vascular smooth muscle cells on standalone electrospun PαAPz-A mats. The presence of elongated and spindle-shaped cells, as well as abundant expression of specific proteins related the vascular smooth muscle cell lineage, demonstrated differentiation. These studies provide strong evidence on the utility of electrospun PαAPz-A as a conducive microenvironment for stem cell differentiation.

## 4. Conclusions

In this study, we first synthesized two PαAPz polymers from L-alanine and L-phenyl alanine. Both PαAPz were electrospinnable and produced beads free fibers with average diameters between 0.1 µm and 0.3 µm. The electrospun fibers from L-phenyl alanine-based PαAPz started to degrade faster than the corresponding PαAPz from L-alanine and became brittle. The PαAPz fibrous mats from L-alanine were able to support adhesion and spreading of iMSC, BM-MSC, and primary human coronary artery SMC. More importantly, electrospun fibers from PαAPz from L-alanine were able to promote differentiation of iMSCs towards SMC lineage. Taken together, our data suggest the utility of PαAPz for differentiating stem cells for tissue engineering applications.

## Figures and Tables

**Table 1:** qRT-PCR primer sequences for human-specific mRNA amplification.

Gene	Forward Primer (5′→3′)	Reverse Primer (3′→5′)
Human *Acta2*	CAA GTG ATC ACC ATC GGA AAT G	GAC TCC ATC CCG ATG AAG GA
Human *Cnn1*	TGA AGC CCC ACG ACA TTT TT	GGG TGG ACT GCA CCT GTG TA
Human *Myh11*	GTC CAG GAG ATG AGG CAG AAA C	GTC TGC GTT CTC TTT CTC CAG C
Human SMTN	CAG GAC AAC AAG GAG AAC TGG	CAG TCA ATT CCT CCA CAT CGT
Human 18S	GCG GTT CTA TTT TGT TGG TTT	CTC CGA CTT TCG TTC TTG ATT

**Table 2:** Comparison of different methods to synthesize PDCP.

Method	Reaction Time at 230 °C	Integration of +20 ppm Peak	Integration of −17 ppm Peak	% Conversion
GS	25 h	1	1.19	54
GS-Ar	54 h	1	6.51	86
FS	58 h	1	5.70	85
FS-Ar	93 h	1	5.32	84
FS-Vacuum	92 h	1	18.91	95
R-FS-Vacuum	92 h	1	15.38	94

GS: grease sealing. GS-Ar: grease sealing with Ar. FS: flame sealing. FS-Ar: flame sealing with Ar. FS-Vacuum: flame sealing under vacuum. R-FS-Ar: recrystallization followed by flame sealing under vacuum.

**Table 3:** Studied electrospinning parameters for PαAPz.

Sample Abbreviations	Solvent Ratio	Distance (cm)	Voltage (kV)	Concentration (wt%)	Flow Rate (mL/h)
**PDCP from FS-vacuum method**					
PαAPz-A	CF:DMSO (3:1)	12–15(*12)	15–20(*20)	7.5–12.5(*10)	0.2–0.6(*0.2)
PαAPz-F	THF:CF (9:1)	9–15(*12)	12–20(*12)	10–15(*10)	0.2–0.6(*0.2)
**PDCP from R-FS-vacuum method**					
PαAPz-A	CF:DMSO (3:1)	12–15(*12)	15–20(*15)	7.5–12.5(*10)	0.2–0.6(*0.2)
PαAPz-A	THF:CF (9:1)	12–15(*12)	15–20(*15)	7.5–12.5(*10)	0.2–0.6(*0.2)
PαAPz-F	THF:CF (9:1)	9–15(*12)	12–20(*12)	10–15(*10)	0.2–0.6(*0.2)

* Optimal electrospinning parameters. CF: Chloroform, THF: tetrahydrofuran, DMSO: dimethylsulfoxide. A—Alanine and F—Phenylalanine.

## Data Availability

The data presented in this study are available on request from the corresponding author.
